# Novel electrochemical PMI marker biosensor based on quantum dot dissolution using a double-label strategy

**DOI:** 10.1038/s41598-022-12444-6

**Published:** 2022-05-25

**Authors:** Bongjin Jeong, Rashida Akter, Jeonghyun Oh, Dong-Gi Lee, Chang-Geun Ahn, Jong-Soon Choi, Md. Aminur Rahman

**Affiliations:** 1 Intelligent Convergence Research Laboratory, Electronics and Telecommunications Research Institute, 34129 Daejeon, Republic of Korea; 2 Graduate School of Analytical Science and Technology, Chungnam National University, 34134 Daejeon, Republic of Korea; 3 Division of Life Science, Korea Basic Science Institute, 34133 Daejeon, Republic of Korea

**Keywords:** Analytical chemistry, Electrochemistry, Biomarkers, Sensors and probes

## Abstract

A novel and facile post-mortem interval (PMI) biosensor was fabricated using a double-label strategy to detect the glyceraldehyde 3-phosphate dehydrogenase (GAPDH) biomarker. A monoclonal anti-GAPDH antibody was immobilized on a surface label containing cadmium selenide quantum dots (CdSe QDs) on a cysteamine graphene oxide (Cys-GO) self-assembled monolayer. Glucose oxidase (GOx) was used as a signal label to conjugate with GAPDH. GAPDH recognition was achieved through the dissolution of the surface-attached CdSe QDs by hydrogen peroxide generated through GAPDH-conjugated GOx-catalyzed *β*-glucose oxidation. To enhance sensitivity, a competitive interaction was introduced between free and conjugated GAPDH to the active site of the anti-GAPDH antibody. The electrochemical response due to CdSe dissolution decreased proportionally with the concentration of free GAPDH. Differential pulsed voltammetry was conducted to determine the analytical characteristics of the immunosensor, including the limit of detection, linear dynamic range, target selectivity, system stability, and applicability toward the analysis of real samples.

## Introduction

Post-mortem interval (PMI) is the time that has elapsed since a person has died. PMI estimation is generally conducted by plain techniques, including livor, algor, and rigor mortis. However, accurate estimation of PMI is essential because it can give important evidence for the investigation of the cause and time of the death. Unfortunately, the accurate determination of PMI is very difficult, requiring many medical/scientific techniques and a long processing time. Therefore, there is an urgent need to develop a simple and rapid method for PMI detection. Glyceraldehyde 3-phosphate dehydrogenase (GAPDH) is a protein, which can be found in saliva and kidney^1^, and its concentration decreases with time after death^2^. This characteristic of the GAPDH protein can be utilized as a suitable protein biomarker for developing a PMI biosensor system.

Various detection methods have been used in biosensor systems involving the antibody-antigen interaction (immunosensors)^3^, such as chemiluminescence^4^, surface plasmon resonance^5^, quartz crystal microbalance^6^, and electrochemical sensing techniques^7^. Among them, the use of an electrochemical immunosensor has attracted significant attention owing to the high sensitivity and selectivity of the sensor^8, 9^. Also, electrochemical immunosensors have shown advantages for protein biomarker detection because of their low cost, easy measurement, fast response, and suitability for point-of-care applications^10–12^^.^ However, the development of an electrochemical immunosensor for PMI detection has been rarely conducted^2^. By using nanomaterials to increase the sensor surface area, this study developed a highly sensitive electrochemical immunosensor to detect GAPDH biomarkers. The PMI immunosensor was manufactured by fixing a monoclonal GAPDH antibody against cadmium selenide (CdSe) quantum dots (QDs), which were attached to the self-assembled monolayer (SAM) of cysteamine containing graphene oxide (GO). GAPDH recognition was achieved through the dissolution of CdSe QDs in hydrogen peroxide^13–15^ generated by the glucose oxidase (GOx)-catalyzed *β*-glucose oxidation^16^^.^ GOx was used as an enzymatic label that was conjugated to the GAPDH protein through glutaraldehyde cross-linking^17^^.^ enhancing the sensitivity, a competitive interaction was introduced between GAPDH-GOx conjugates and free GAPDH with the active site of anti-GAPDH^18^^.^ The current reaction due to CdSe dissolution decreased proportionally to the increased free-GAPDH concentration. Thus, it was possible to quantify free GAPDH with this strategy, and differential pulsed voltammetry (DPV) was conducted to determine the analytical characteristics of an immunosensor, including the limit of detection, linear dynamic range, target selectivity, system stability, and applicability toward the analysis of real samples^19^^.^

## Materials and methods

### Reagents and solutions

Cysteamine, graphene oxide (2 mg/mL, dispersion in H_2_O), N-(3-dimethylaminopropyl)-N’-ethylcarbodiimide (EDC), N-hydroxysulfosuccinimide sodium salt (NHS), sodium phosphate dibasic, sodium phosphate monobasic, glutaraldehyde solution, sodium chloride, Sephadex G-25, glucose oxidase (from Aspergillus niger), *β*-D-glucose pentaacetate, ethylene diamine tetraacetic acid (EDTA), trizma (NH_2_C(CH_2_OH)_3_·HCl) (Tris), bovine serum albumin (BSA), prostate specific antigen (PSA), carcinoembryonic antigen (CEA), c-reactive protein (CRP) human immunoglobulin G (hIgG), horseradish peroxidase (HRP), and human serum samples were purchased from Sigma-Aldrich Co. Hydrochloric acid was purchased from Junsei Co., and human *α*-thrombin was obtained from Haematologic Technologies Inc. The GAPDH antibody (Sc-25778) was obtained from Santa Cruz Co., and the GAPDH solution (concentration: 10.532 mg/mL) was obtained from Cosmogenetech Co., South Korea. The QDs (CdSe/ZnS with carboxylic acid active group in water, 520 nm emission/450 nm absorption) were purchased from Global Zeus Co., South Korea. All chemicals were of analytical grade and used without further modification. All aqueous solutions were prepared using deionized (DI) water from a Milli-Q water purification system (18 MΩ·cm). The electrochemical evaluations were conducted in 0.1 mM phosphate buffer solution (PBS, pH 7.4). All solutions were deoxygenated by 99.9%-purity nitrogen for more than 15 min.

### Apparatus

Electrochemical evaluation techniques such as DPV and cyclic voltammetry (CV) were measured through a potentiostat/galvanostat (CHI660D, CH Instruments Inc, USA). For DPV, Ag/AgCl (saturated KCl) and platinum (Pt) were used as reference and counter electrodes, respectively. Scanning electron microscopy (SEM) and energy-dispersive X-ray spectroscopy (EDS) were conducted on an SEM system (CLARA, TESCAN, Czech Republic). Confocal microscope images were obtained from a confocal scanning microscope (LSM 880 with Airyscan, Carl zeiss, Germany). Ultraviolet-visible (UV-vis) spectroscopy was performed through a UV-vis spectrophotometer (Optizen POP, Mecasys, Korea Republic).

### Fabrication of immunosensor probe

**Figure 1:**
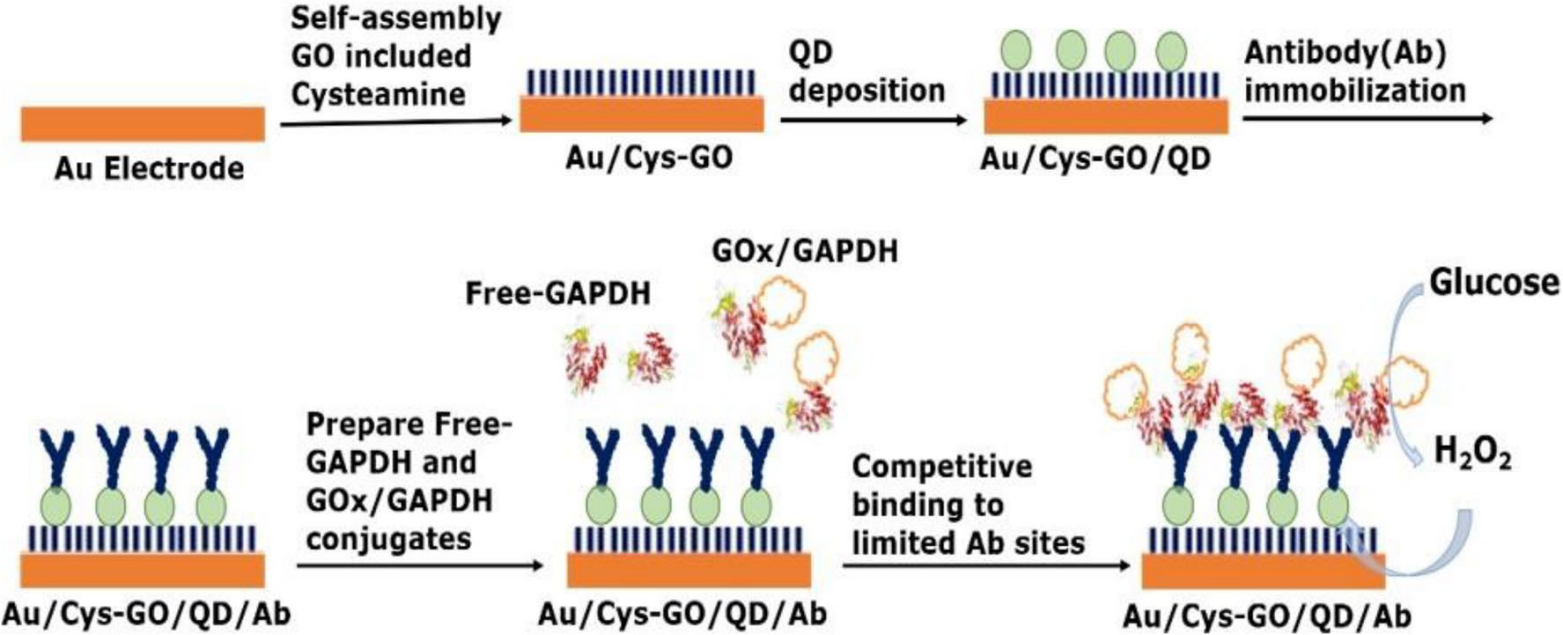
Schematic of GAPDH detection by using the QD-dissolution immunosensor.

The immunosensor platform was modified by GO-incorporated SAM of cysteamine (Cys) through chemical binding^20^. First, Au electrodes were polished with a 0.05 *µ*m alumina/water slurry on a polishing cloth. Next, the polished electrode was sonicated and rinsed by DI water. Then, the mirror-finished electrode was washed out with a piranha solution (70% H_2_SO_4_ and 30% H_2_O_2_), rinsed completely with DI water, and dried in an oven. The Cys-GO solution was prepared by mixing 36 mM Cys in DI water with 10 *µ*g/mL GO. The final mixture (5 *µ*L) was dropped on the Au electrode and kept for 8 h^21, 22^ for self-assembly. The carboxylic acid groups on QDs were activated with 10 mM EDC/NHS solution^23^, and the Au/Cys-GO-modified surface was covalently bonded with the activated QDs for 10 h (Au/Cys-GO/QD)^24^. After washing, the Au/Cys-GO/QD electrode was incubated in a 0.1 M PBS solution (pH 7.0) containing 1 *µ*g/mL anti-GAPDH for 4 h^25–27^^.^ The GAPDH antibody (Ab) was immobilized on the QDs via the amide bonds between the carboxyl group of the QD and the amine group of anti-GAPDH (Au/Cys-GO/QD/anti-GAPDH). After that, the electrode was washed three times using 0.1 M PBS, then placed in 0.05% BSA solution for 1 h to block the non-specific absorption of other proteins. After rinsing three or four times using PBS, the BSA-blocked Au/Cys-GO/QD/anti-GAPDH immunosensor was stored in the refrigerator at 4 °C. Figure 1 describes the overall fabrication of the GAPDH immunosensor.

### Preparation of GOx/GAPDH conjugates

The GOx/GAPDH conjugates were prepared according to the following procedure^28, 29^. First, 50 *µ*g/mL of GAPDH in 1.0 M PBS and 0.5 mL of 25% glutaraldehyde were mixed at room temperature (RT) for 18 h (glu-GAPDH). Excess glutaraldehyde was filtered by a Sephadex G-25 column in 10 mM tris/1 mM EDTA (pH 7.5) equilibrated with 0.9% NaCl. Then, 3 mL GOx (1.0 mg/mL) in 1 M PBS solution was mixed with 6 mL of glu-GAPDH to induce covalent cross-linking between GOx and glu-GAPDH. The mixture was incubated for 24 h at RT (conjugated GAPDH). In order to prevent the non-specific binding of the remaining active site of the prepared conjugate, blocking was performed using 0.1% (w/v) BSA solution, and dialysis was performed in 0.1 M PBS (pH 7.4). Finally, the conjugate was filtered through a sterile Millipore membrane (0.20 *µ*m) then stored at  −  20 °C.

## Results and discussion

### SEM, EDS, confocal microscopy, XPS, CV of the immunosensor probe

**Figure 2:**
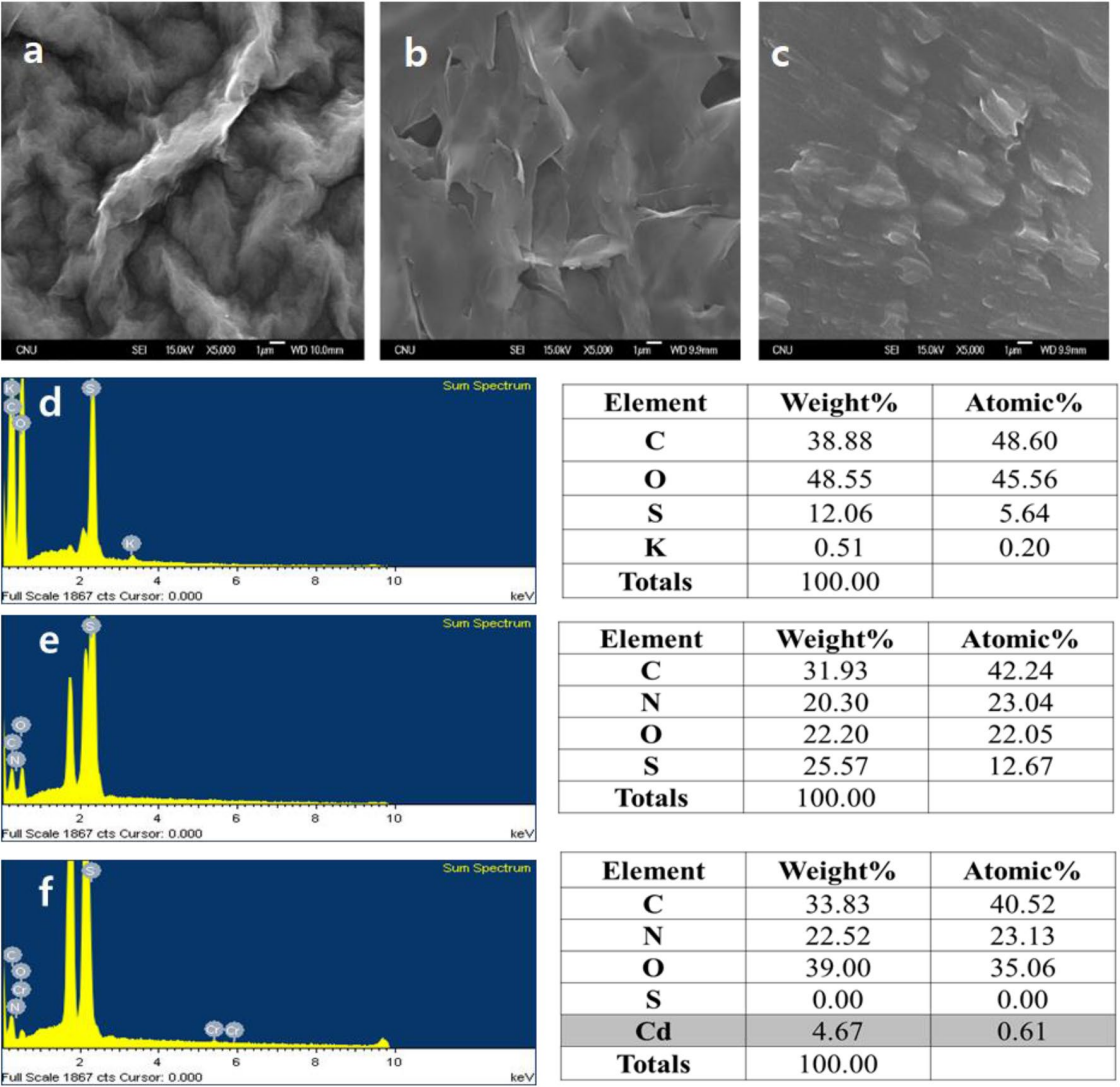
SEM images of (**a**) GO, (**b**) Cys-GO and (**c**) Cys-GO/QD modified surfaces. (**d**) GO, (**e**) Cys-GO and (**f**) Cys-GO/QD EDS spectra obtain for Au/GO (i), Au/Cys-GO (ii) and Au/Cys-GO/QD (iii) modified surfaces.

Figure 2 shows the SEM images of GO-, Cys-GO-, and Cys-GO/QD-modified Au surfaces. The GO electrode exhibited a thick and rugged morphology in the SEM image. For the Cys-GO electrode, a big spot was observed on the smooth SAM surface, confirming that GO was incorporated in Cys. The Cys-GO/QD-modified Au surface showed some offshoots due to QD attachment after QD binding. However, the QDs are small nanoparticles of 5 nm size; thus, they are not visible in the images.

EDS was performed to characterize the surface of the Au/Cys-GO/QD electrode (Fig. 2). The survey spectra of Au/GO and Au/Cys-GO indicated the presence of C, O, S, and K and C, N, O, and S, respectively. However, the Au/Cys-GO/QD electrode was found to possess C, N, O, S and Cd. The presence of Cd on the surface indicated the successful immobilization of the QDs on the immunosensor.

XPS analysis was performed in order to characterize the covalent bonding of QD with Au/Cys-GO as shown in Fig. S1. The C1S peak in Au/Cys-GO/QD and the N1S peak in Au/Cys-GO shifted to higher and lower energies, respectively, clearly indicates the formation of the covalent bonding between the carboxylic acid of QD and the amine groups of cysteamine in Au/Cys-GO.

Confocal microscopy was conducted to further characterize the GAPDH immunosensor surface. Fig. S2 shows the fluorescence images of Au/Cys and Au/Cys-GO/QD. The fluorescence was evident from the Au/Cys-GO/QD electrode surface owing to QD emission, whereas the Au/Cys electrode did not exhibit any fluorescence. These results confirm that the QDs have been successfully immobilized on the Au/Cys-GO surface.

The electrochemical behavior of the Au/Cys-GO/QD electrode was analyzed by CV. Fig. S3 shows the CV profiles of the Au/Cys-, Au/Cys-GO-, and Au/Cys-GO/QD-modified electrodes in PBS. A very small redox peak was observed for the Au/Cys-modified electrode because of the absence of electroactive materials such as nanoparticles for the redox reaction. Moreover, for the Au/Cys-GO-modified electrode, a significantly increased redox peak between −0.1 V and 0.3 V vs. Ag/AgCl was observed, suggesting that the introduction of GO improved the conductivity and increased the maximum current. After Au/Cys-GO/QD modification, the redox peak was located at the same position as that observed for the Au/Cys-GO electrode, with an increment in the peak current. The separation between the reduction and oxidation peaks was calculated as 0.4 V, implying a quasi-reversible electron transfer reaction**.** Table S1 shows the increase rate of the redox peak, indicating a gradual increase in current following further steps. Theses analysis results confirmed that Cys-GO/QD was successfully immobilized on the Au electrode surface with an electroactive characteristic and can be used for the fabrication of the GAPDH immunosensor.

### UV/Vis characteristics of the QD dissolution

The QD dissolution was analyzed by UV/Vis spectroscopy. Fig. S4 shows the UV/Vis spectra obtained for 10 *µ*g/ml QDs, 10 *µ*g/ml QDs reacting with 5% H_2_O_2_ for 5 min, and 10 *µ*g/ml QDs reacting with 1% H_2_O_2_ for 5 min. As expected, the QDs showed a peak between 440 and 460 nm. After the QDs reacted with H_2_O_2_, the absorbance was remarkably reduced; the high concentration of H_2_O_2_ afforded lower absorbance than the low concentration, confirming that the QD dissolution was related to the presence of H_2_O_2_.

### Optimization

To achieve the best response in real sample analysis, the dilution ratio of the antibody was optimized. Fig. S5 shows the response of the anti-GAPDH antibodies to the detection probes diluted in various ratios from 1:2000 to 1:40 under 1 ng/mL free GAPDH conditions. The current response increased significantly when the Ab dilution factor varied from 1:2000 to 1:200 and did not increase further when the dilution factor was 1:200 or higher. Thus, the optimum anti-GAPDH antibody ratio was finalized as 1:200. The pH and incubation time optimizations are important for maximum sensitivity in antibody-antigen interaction based immunosensor. However, we did not try to optimize the pH and incubation time in this study because our aim was to use this immunosensor at a physiological pH condition (pH=7.0 ~ 7.4). Regarding incubation time of antibody-antigen interaction, previous results on antibody-antigen binding showed 30 min −  2 h time incubations appropriate for maximum responses^25–27^^.^. Considering the fact that most of antibody-antigen interactions are needed to have similar binding time, we did not attempt to optimize it. However, for the maximum antibody-antigen binding, we used 4 h incubation time as the incubation step was performed at 4 ℃.

### Analytical performance of GAPDH immunosensor

**Figure 3:**
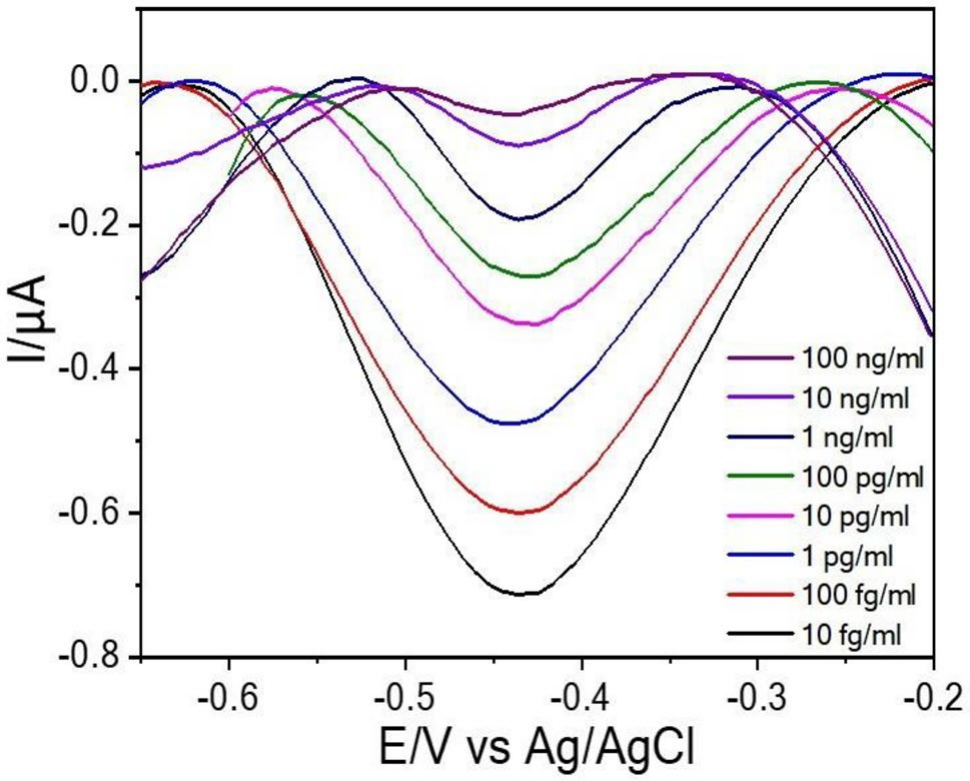
DPV responses obtained for the immunosensor at various concentration of free GAPDH.

DPV is a more sensitive technique than CV; thus, it was used for the quantification of free GAPDH. Figure 3 shows the DPV responses measured after the dissolution of QDs at various concentrations of free GAPDH ranging between 10 fg/mL and 100 ng/mL. The response was measured after incubating the immunosensor in PBS containing conjugated GAPDH and various concentrations of GAPDH for 10 h. As shown in Fig. 3, a stripping peak at −0.43 V vs. Ag/AgCl was observed owing to the dissolution of Cd to Cd^+^. Next, the immunosensor was placed with both conjugated GAPDH and free GAPDH in PBS. Free GAPDH competed for binding to the active sites at the anti-GAPDH antibody; thus, the amount of conjugated GAPDH bound to the antibody active sites was reduced and, consequently, the reduction current peak of Cd stripping decreased due to the presence of free GAPDH. Because the bounded GOx interacting with *β*-glucose in PBS solutions generated H_2_O_2_^30^, the enzymatically generated H_2_O_2_ could dissolve Cd metal of QDs. Therefore, the Cd stripping peak gradually decreased and its intensity was proportional to the amount of free GAPDH.

In order to achieve sensitive GAPDH detection, the analytical performance was carried out under optimized experimental conditions. When the concentration of free GAPDH was increased and the amount of conjugated GAPDH was fixed, a low amount of GOx coupled with the antibody binding sites via the competitive binding strategy^31^, generating a low amount of H_2_O_2_ for QD dissolution. As a result, the intensity of the Cd stripping peak did not significantly decrease. The response of stripping current increased upon the decrease in the concentration of free GAPDH in PBS and a direct linear relationship was observed between the current response and free-GAPDH concentration, enabling the quantification of free GAPDH by this dissolution strategy.

**Figure 4:**
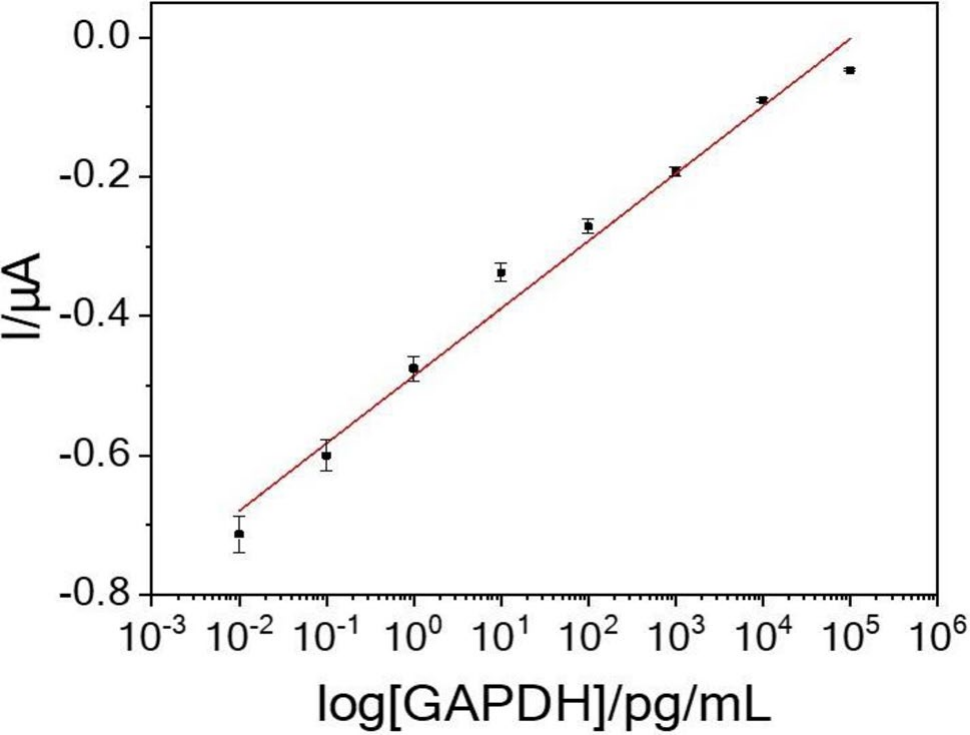
Calibration plot using the stripping-current response.

Figure 4 shows the calibration plot using the stripping-current intensity obtained from Fig. 3. The linear range of GAPDH detection was determined to be from 10 fg/mL to 100 ng/mL. The linear dependence between the intensity and free-GAPDH concentration produced a regression equation of I (y) = 0.0969 · log x + 0.7769 with a correlation coefficient of 0.9802. The relative standard deviation was approximately 5.16% (n=5) at a GAPDH concentration of 1 ng/mL. Based on three measurements of the standard deviation of the blank noise (95% confidence level, k = 3, n = 5), the limit of detection was determined as 2 fg/mL. All analytical parameters are summarized in Table S2. The analytical parameters obtained in this study and those of previously reported QD-based biosensors are compared in Table 1^32–38^, demonstrating the high sensitivity of the GAPDH sensor developed in this work.

**Table 1:** Comparison with other QDs biosensors.

	Role of QDs	Target	Detection method	Detection limit
1^32^	Colloidal QDs modification layer	COVID-19	Electrochemical	4.99 ng/mL
2^33^	CdSe/ZnS QDs labeled conjugate	Salmonella	Fluorescent	4.9 × 10^3^ cfu/mL
3^34^	Graphene QDs modified layer	Thrombin	Electrochemical	100 nM
4^35^	Graphene QDs enzymatic label	Glucose	Electrochemical	3.38 μM
5^36^	CA-capped CdTe QDs as functional molecules	Glutathione	Fluorescent	3.3 nM
6^37^	Graphene QDs enhancing label	MMP-2	Electrochemiluminescence	6.5 pg/mL (0.09 pM)
7^38^	Carbon dots as amplification label	Cocaine	Electrochemical	0.26 pM
This work	CdSe QDs for conductive layer and amplification signal	GAPDH	Electrochemical	2.00 fg/mL (0.06 fM)

### Selectivity, stability, and real sample analysis

**Figure 5:**
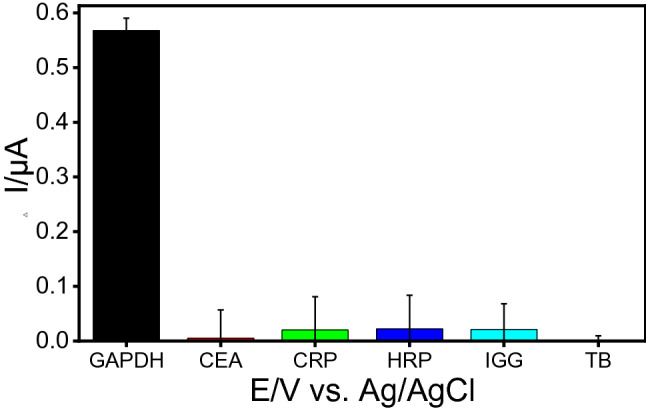
Selectivity study of the GAPDH electrochemical immunosensor in the presence of other proteins (CEA, CRP, HRP, IGG, TB) under 1 ng/mL conditions.

To investigate the selectivity, the competitive binding of the immunosensor was investigated using conjugated GAPDH and similar biomarkers such as PSA, carcinoembryonic antigen (CEA) CRP human immunoglobulin G (hIgG), horseradish peroxidase (HRP), and thrombin (TB). Figure 5 shows the DPV responses obtained before and after a competitive reaction. Except GAPDH, all tested proteins did not show any significant current changes after the competition, suggesting that the proposed immunosensor is highly selective and the above-mentioned proteins did not interfere with GAPDH detection.

**Figure 6:**
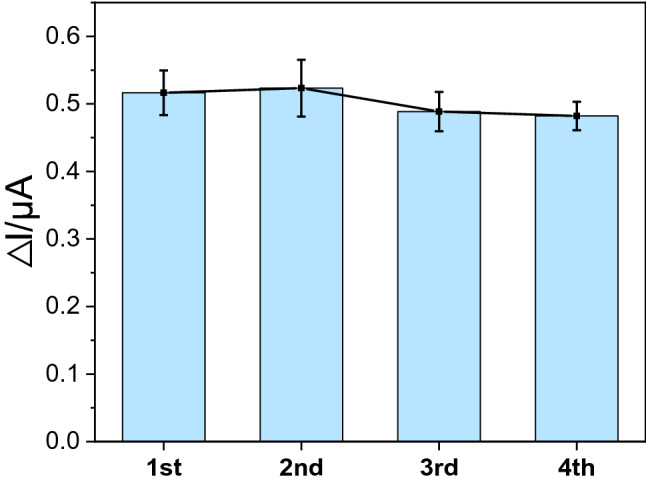
Current gap value of 100 pg/mL of GAPDH tested on different days.

The stability of the fabricated sensor was determined by measuring the response of 100 pg/mL of GAPDH for one month. After each measurement, the immunosensor was washed with a PBS solution and dipped into a 0.2 M Gly-HCl buffer solution (pH=2.8) for 5 min. After three times washing with a PBS solution, the immunosensor was stored in a dry condition at 4 °C until it was further used for competitive binding of free and conjugated GAPDH. The current response did not find to be significantly decreased (0.516, 0.523, 0.488, and 0.482) for one month as shown in Fig. 6. The immunosensor retained almost 93.4 % of its initial response for one month time period. These results indicated that the GAPDH immunosensor exhibited not only high selectivity but also long-term stability.

**Figure 7:**
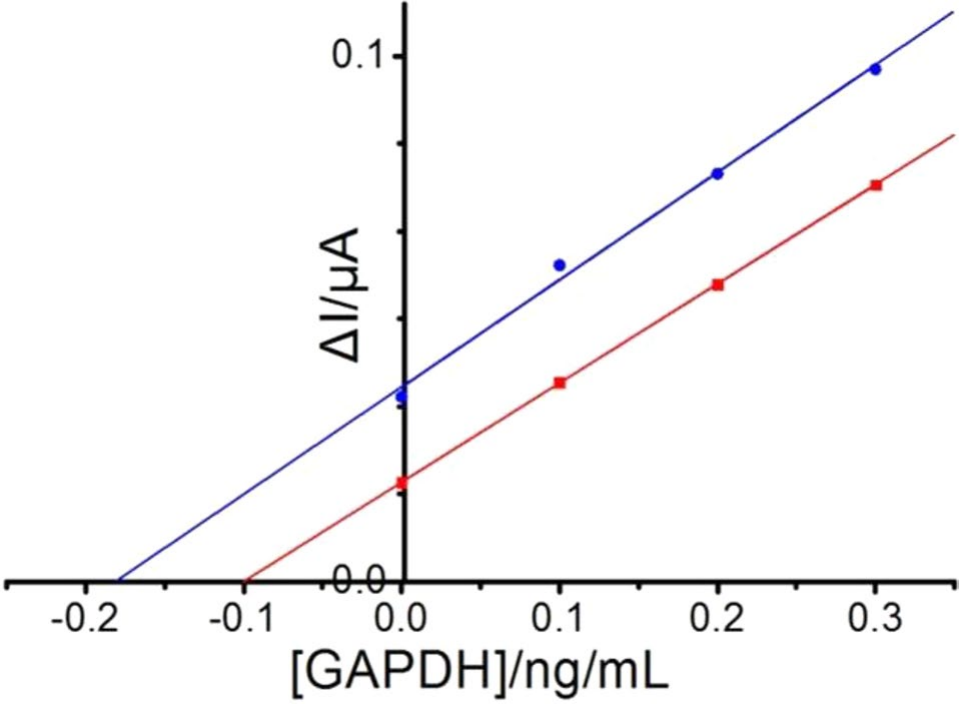
Standard addition plot for GAPDH detection in real human serum samples.

The applicability of this GAPDH immunosensor for the analysis of real samples was investigated by using human blood serum. For this purpose, blood serum samples were prepared by spiking 0.1 ng/mL ±0.03 and 0.2 ng/mL GAPDH. To calculate the GAPDH concentration, the standard addition method was used; the obtained plot is shown in Fig. 7. The concentration of GAPDH was detected at 0.1 ±0.03 and 0.19 ±0.03 ng/mL. The recovery was almost 100% for the first sample and 95% for the second sample, indicating acceptable accuracy. This result confirms that the proposed GAPDH immunosensor can be applied for GAPDH detection in real human serum samples.

## Conclusions

We developed an electrochemical immunosensor based on Cys-GO/QD platform to detect the GAPDH biomarker for PMI estimation. The Cys-GO/QD layer has the characteristics of a large surface area and excellent biocompatibility, which enable the immobilization of GAPDH antibodies. The co-existence of GO and QD amplified the electrochemical signal by enhancing the conductivity and generating numerous electrons for detection. More importantly, metallic stripping of QDs was induced by H_2_O_2_ generated from enzymatic labels, and this novel sensing strategy further improved the detection sensitivity. The developed GAPDH immunosesnsor can detect GAPDH at a low concentration of 2.0 fg/mL with a broad linear scope between 1.0 fg/mL and 100 ng/mL. The proposed detection strategy can be used in forensic field for the point-of-care detection of GAPDH and other PMI biomarkers and is promising for PMI estimation.

## Supplementary Information

Supplementary Information.

## Data Availability

No datasets were generated or analyzed during the current study.

## References

[1] Inoue H, Kimura A, Tuji T (2002). Degradation profile of mRNA in a dead rat body: basic semi-quantification study. Forensic Sci. Int..

[2] Lee D-G (2016). Degradation of kidney and psoas muscle proteins as indicators of post-mortem interval in a rat model, with use of lateral flow technology. PLoS ONE.

[3] Turner AP (1997). Immunosensors: The next generation. Nat. Biotechnol..

[4] Jie G, Li L, Chen C, Xuan J, Zhu J-J (2009). Enhanced electrochemiluminescence of CdSe quantum dots composited with CNTs and PDDA for sensitive immunoassay. Biosens. Bioelectron..

[5] Scarano S, Mascini M, Turner APF, Minunni M (2010). Surface plasmon resonance imaging for affinity-based biosensors. Biosens. Bioelectron..

[6] Tan Y (2010). Immobilization of enzymes at high load/activity by aqueous electrodeposition of enzyme-tethered chitosan for highly sensitive amperometric biosensing. Biosens. Bioelectron..

[7] Heineman WR, Halsall HB (1985). Strategies for electrochemical immunoassay. Anal. Chem..

[8] Du D, Xu X, Wang S, Zhang A (2007). Reagentless amperometric carbohydrate antigen 19–9 immunosensor based on direct electrochemistry of immobilized horseradish peroxidase. Talanta.

[9] Prabhulkar S, Alwarappan S, Liu G, Li C-Z (2009). Amperometric micro-immunosensor for the detection of tumor biomarker. Biosens. Bioelectron..

[10] Zhang B (2012). DNA-based hybridization chain reaction for amplified bioelectronic signal and ultrasensitive detection of proteins. Anal. Chem..

[11] Lv S (2019). H_2_-based electrochemical biosensor with Pd nanowires@ZIF-67 molecular sieve bilayered sensing interface for immunoassay. Anal. Chem..

[12] Gao Y, Zeng Y, Liu X, Tang D (2022). Liposome-mediated *in situ* formation of Type-I heterojunction for amplified photoelectrochemical immunoassay. Anal. Chem..

[13] Numnuam A (2008). Aptamer-based potentiometric measurements of proteins using ion-selective microelectrodes. Anal. Chem..

[14] Thürer R (2007). Potentiometric immunoassay with quantum dot labels. Anal. Chem..

[15] Hansen JA (2006). Quantum-dot/aptamer-based ultrasensitive multi-analyte electrochemical biosensor. J. Am. Chem. Soc..

[16] Wong CM, Wong KH, Chen XD (2008). Glucose oxidase: natural occurrence, function, properties and industrial applications. Appl. Microbiol. Biotechnol..

[17] Walt DR, Agayn VI (1994). The chemistry of enzyme and protein immobilization with glutaraldehyde. TrAC Trends Anal. Chem..

[18] Jeong B, Han O-H, Rhee CK (2013). Multiple nonenzymatic labels-based impedimetric aptamer sensor for the competitive detection of thrombin. Bull. Korean Chem. Soc..

[19] García-Armada P, Losada J, de Vicente-Pérez S (1996). Cation analysis scheme by differential pulse polarography. J. Chem. Educ..

[20] Kerekovic I, Milardovic S, Palcic M, Grabaric Z (2014). Characterization of cysteamine self assembled on gold functionalized with nitrilotriacetic acid and evaluation of copper(II) binding capacity with adsorption transfer stripping voltammetry. J. Electroanal. Chem..

[21] Wirde M, Gelius U, Nyholm L (1999). Self-assembled monolayers of cystamine and cysteamine on gold studied by XPS and voltammetry. Langmuir.

[22] Shervedani RK, Farahbakhsh A, Bagherzadeh M (2007). Functionalization of gold cysteamine self-assembled monolayer with ethylenediaminetetraacetic acid as a novel nanosensor. Anal. Chim. Acta.

[23] Pattabiraman VR, Bode JW (2011). Rethinking amide bond synthesis. Nature.

[24] Akter R, Rahman MdA, Rhee CK (2012). Amplified electrochemical detection of a cancer biomarker by enhanced precipitation using horseradish peroxidase attached on carbon nanotubes. Anal. Chem..

[25] Akter R, Jeong B, Lee Y-M, Choi J-S, Rahman MdA (2017). Femtomolar detection of cardiac troponin I using a novel label-free and reagent-free dendrimer enhanced impedimetric immunosensor. Biosens. Bioelectron..

[26] Akter R, Jeong B, Choi J-S, Rahman MdA (2016). Ultrasensitive Nanoimmunosensor by coupling non-covalent functionalized graphene oxide platform and numerous ferritin labels on carbon nanotubes. Biosens. Bioelectron..

[27] Biswas S, Lan Q, Li C, Xia X-H (2022). Morphologically flex Sm-MOF based electrochemical immunosensor for ultrasensitive detection of a colon cancer biomarker. Anal. Chem..

[28] Tijssen, P. *Practice and theory of enzyme immunoassays*. (Elsevier ; Sole distributors for the USA and Canada, Elsevier Science Pub. Co., 1985).

[29] Akter, R., Kyun Rhee, C. & Rahman, Md. A. A stable and sensitive voltammetric immunosensor based on a new non-enzymatic label. *Biosens. Bioelectron.* **50**, 118–124 (2013). 10.1016/j.bios.2013.06.016 23845689

[30] Raba J, Mottola HA (1995). Glucose oxidase as an analytical reagent. Crit. Rev. Anal. Chem..

[31] Jeong B, Akter R, Choi J-S, Rahman MdA (2015). Highly sensitive voltammetric thrombin aptamer sensor based on the synergistic effect of doping/depositing gold nanoparticles in polydopamine film. Electroanalysis.

[32] Zhao Y (2022). All-solid-state SARS-CoV-2 protein biosensor employing colloidal quantum dots-modified electrode. Biosens. Bioelectron..

[33] Ding S (2022). A fluorescent biosensor based on quantum dot–labeled streptavidin and poly-l-lysine for the rapid detection of Salmonella in milk. J. Dairy Sci..

[34] Zhao J, Chen G, Zhu L, Li G (2011). Graphene quantum dots-based platform for the fabrication of electrochemical biosensors. Electrochem. Commun..

[35] Zhang Y (2022). Cascade amplification based on PEI-functionalized metal–organic framework supported gold nanoparticles/nitrogen–doped graphene quantum dots for amperometric biosensing applications. Electrochim. Acta.

[36] Zhu Q (2022). Highly selective and sensitive detection of glutathione over cysteine and homocysteine with a turn-on fluorescent biosensor based on cysteamine-stabilized CdTe quantum dots. Spectrochim. Acta. A. Mol. Biomol. Spectrosc..

[37] Fan X (2022). A sensitive electrochemiluminescence biosensor for assay of cancer biomarker (MMP-2) based on NGQDs-Ru@SiO2 luminophore. Talanta.

[38] Azizi S, Gholivand MB, Amiri M, Manouchehri I, Moradian R (2022). Carbon dots-thionine modified aptamer-based biosensor for highly sensitive cocaine detection. J. Electroanal. Chem..

