# Supplementary material for: Novel electrochemical PMI marker biosensor based on quantum dot dissolution using a double-label strategy
Source: Sci Rep. 2022 May 25;12:8815. doi: 10.1038/s41598-022-12444-6 (PMC9130975; doi:10.1038/s41598-022-12444-6)
Supplement: Supplementary file 1 — Supplementary Information. [file 41598_2022_12444_MOESM1_ESM.docx]

**Supplementary Information**

**Novel Electrochemical PMI Marker Biosensor Based on Quantum Dot Dissolution Using a Double Label Strategy**

Bongjin Jeong^1,2*+^, Rashida Akter^2+^, Jeonghyun Oh^2^, Dong-Gi Lee^3^, Chang-Geun Ahn^1^, Jong-Soon Choi^2,3*^, and Md.Aminur Rahman^2,*^

^1^Intelligent Convergence Research Laboratory, Electronics and Telecommunications Research Institute, 34129, Daejeon, Republic of Korea

^2^Graduate School of Analytical Science and Technology, Chungnam National University, 34134, Daejeon, Republic of Korea

^3^Division of Life Science, Korea Basic Science Institute, 34133, Daejeon, Republic of Korea


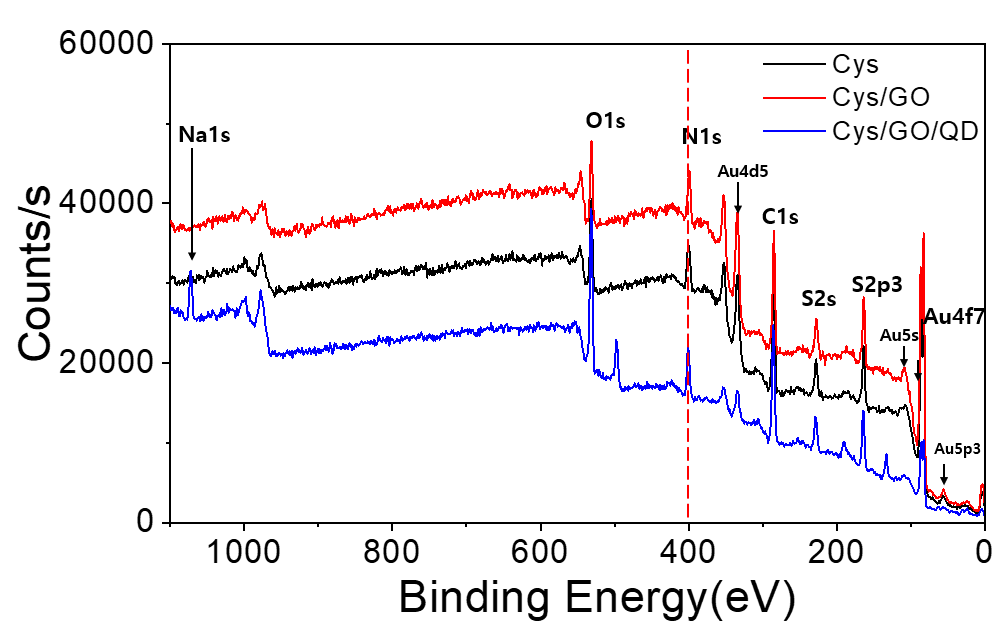


**Fig. S1. XP spectra of Cys, Cys/GO, and Cys/GO/QD**


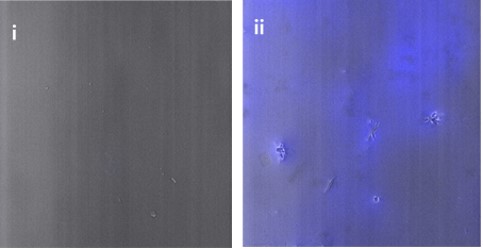


**Fig. S2.** Confocal microscope images of Cys-GO(i), Cys-GO/QD(ii).


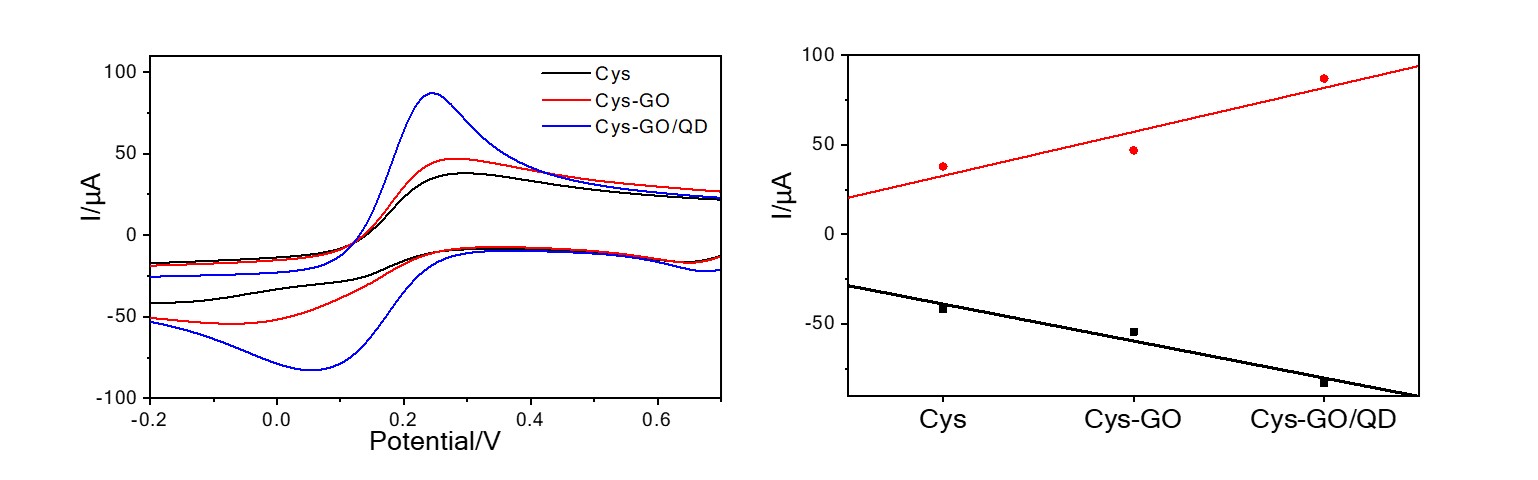


**Fig. S3.** CV responses of Cys and Cys-GO and Cys-GO/QD modified electrode (a), Plot of anodic (oxidation) and cathodic (reduction) peak currents rate for Cys-GO/QD modified electrode (b).


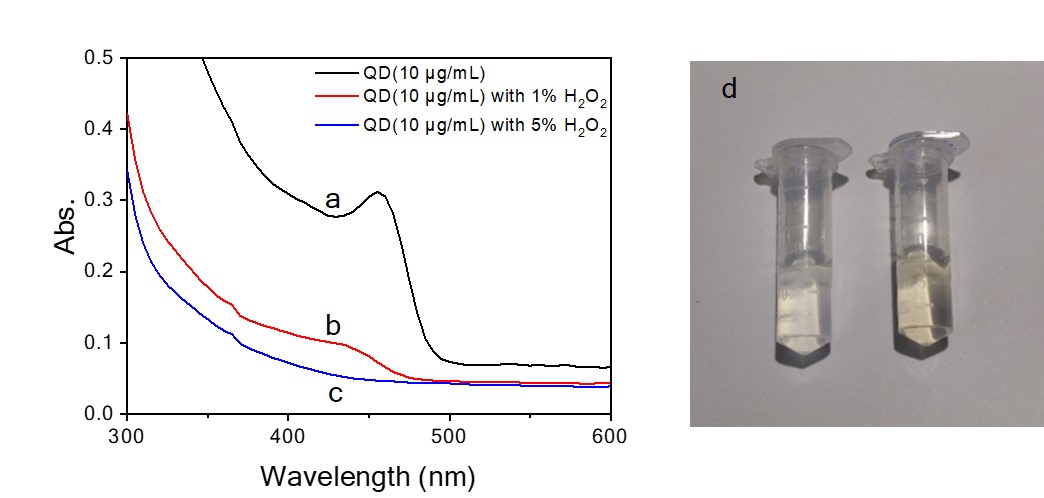


**Fig. S4.** UV Spectrum of QD (a), 10 g/mL QD react with 1% H_2_O_2_ (b), QD react with 5% H_2_O_2_ (c) during 10 minutes and picture of 10 μg/mL QD react with 5% H_2_O_2_ (left) , 10 μg/mL QD(right) (d).


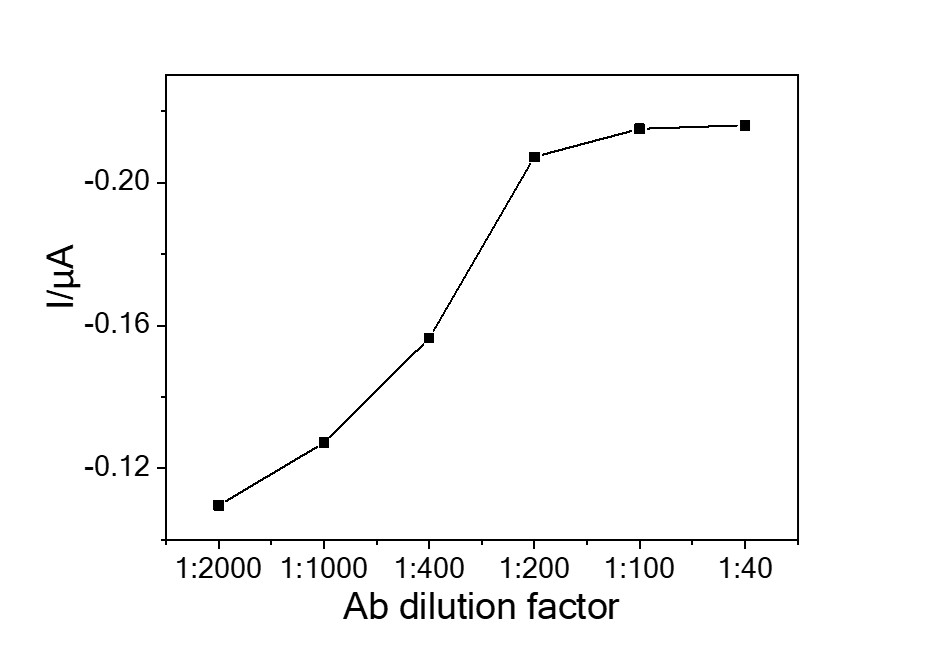


**Fig. S5.** The effects of the concentration of an antibody during immobilization.

| Parameter | Step 1-2 | Step 2-3 |
| --- | --- | --- |
| Reduction increase rate | 31% | 52% |
| Oxidation increase rate | 24% | 85% |

**Table S1.** The oxidation reduction potential increase rate of modified electrode.

| Parameter | Value |
| --- | --- |
| R^2^ Value | 0.9802 |
| Correlation Equation | Y=0.0969·X-0.7769 |
| Detection Limit | 2 fg/mL |
| Standard Deviation | 5.16% |
| Linear Range | 10 fg/mL to 100 ng/mL |

**Table S2.** Analytical parameters of GAPDH immunosensor.
